# Rainbow trapping in a chirped three-dimensional photonic crystal

**DOI:** 10.1038/s41598-017-03454-w

**Published:** 2017-06-08

**Authors:** Zeki Hayran, Hamza Kurt, Kestutis Staliunas

**Affiliations:** 1 Nanophotonics Research Laboratory, Department of Electrical and Electronics Engineering, TOBB University of Economics and Technology, Ankara, 06560 Turkey; 2 DONLL, Departament de Física, Universitat Politècnica de Catalunya (UPC), Edifici Gaia, Rambla Sant Nebridi 22, 08222 Terrassa, Spain; 3 Institució Catalana de Recerca i Estudis Avancats (ICREA), Passeig Lluís Companys 23, 08010 Barcelona, Spain

## Abstract

Light localization and intensity enhancement in a woodpile layer-by-layer photonic crystal, whose interlayer distance along the light propagation direction is gradually varied, has been theoretically predicted and experimentally demonstrated. The phenomenon is shown to be related to the progressive slowing down and stopping of the incident wave, as a result of the gradual variation of the local dispersion. The light localization is chromatically resolved, since every frequency component is stopped and reflected back at different positions along the crystal. It has been further discussed that the peculiar relation between the stopping position and the wave vector distribution can substantially increase the enhancement factor to more than two orders of magnitude. Compared to previously reported one- and two-dimensional photonic crystal configurations, the proposed scheme has the advantage of reducing the propagation losses by providing a three-dimensional photonic bandgap confinement in all directions. The slowing down and localization of waves inside photonic media can be exploited in optics and generally in wave dynamics, in many applications that require enhanced interaction of light and matter.

## Introduction

Temporary storage of light, by means of its slowing down which leads to increase of the local intensity of slow light, has been the subject of intensive research, since many useful optical phenomena rely on strong interaction between light and matter^1^. Initial attempts to substantially reduce the speed of light were based on quantum effects such as electromagnetic induced transparency^2^ and optical coherent effects^3^, where restrictions like the usage of ultracold gases or the narrow operational bandwidth limited their practical implementations. More recently the on-chip realizations of slow light schemes have become possible, such as coupled resonator optical waveguides^4, 5^, which can lead to low group velocities with negligible distortion. However a fundamental limit, limiting such structures is the so-called delay-bandwidth product, which imposes a trade-off between the factor of slowing-down and the bandwidth in which the slowing, exists^6^. One way to alleviate this limitation is to adiabatically tune (or chirp) the structure, meaning that one or several structural parameters are gradually varied along the direction of propagation. In particular, it has been shown that in a tapered metamaterial heterostructure, light can be slowed down and trapped at specific locations depending on its frequency using negative Goos–Hänchen shift^7^. This phenomenon, termed as the “Trapped Rainbow” effect, can also be considered as the spatial separation of the frequency components of the propagating wave, and has been demonstrated in dielectric^8^ and plasmonic^9–18^ gratings or waveguides, in one-^19^ and two-dimensional^20–22^ photonic crystals (2D PhCs), in a hyperbolic metamaterial waveguide^23^, in sonic crystals^24^, and in a waveguide under a tapered magnetic field^25^. While significant steps have been taken towards the slowing down and “trapping” of the light, one still needs to cope with extrinsic losses while implementing such schemes. It has been debated whether material absorption that is reminiscent of metallic structures can give large propagation losses and prevent the light stopping mechanism in metamaterials^26^. Although it is possible to overcome the material losses in metamaterials by employing absorption-free dielectric 2D PhCs in their negative refraction regime^27^ instead of metallic components, it still remains unclear whether other extrinsic loss mechanisms such as out-of-plane radiation losses can be compensated and overcome in such structures. Yet some attempts to reduce the losses in 2D PhCs have been undertaken^28, 29^, the inevitable relation between the group velocity of the light and its interaction time with the medium still leads to considerable amount of scattering loss enhancement, as one approaches the band edge region (where most of the slow light behavior occurs)^27, 30–33^.

In contrast to their 2D counterparts, three-dimensional (3D) PhCs are capable of providing complete photonic bandgaps (PBGs) in all three directions. Among them, the layer-by-layer (or namely the woodpile) PhCs have been intensively studied due to their flexible micro- or nanofabrication requirements and their robustness against fabrication imperfections^34, 35^. Such a structure consists of layers of parallel rods or logs, where the rods in each consecutive layer are rotated by 90°. Additionally, each layer with the same orientation of rods is shifted relative to each other by a half of the in-plane period (*a*), forming a 3D PhC of the symmetry of a face-centered tetragonal lattice. In this study, we propose a woodpile PhC with gradually varied longitudinal periods in the stacking direction, to gradually slow down and stop the incident wave. We first present the local dispersion analysis of such a configuration which suggest the choice of structural parameters. We then evaluate the intensity enhancement factors and calculate the spatial positions where the wave stops and the maximal field enhancement occurs, by means of instantaneous and steady-state field distribution analysis. Finally we verify the principle experimentally in the microwave regime, by comparing the numerically calculated and experimentally measured field profiles and the intensity enhancement factors.

We note that various “trapped rainbow” guiding schemes have been already experimentally demonstrated at microwave^12–14^ or even at visible^16, 18, 36, 37^ frequencies. However in such waveguide or grating based structures, the modal volume of the localized wave is inevitably restricted by the volume of the defect waveguide or the width of the tapered grating. Such a limitation impose severe constraint onto the local field intensity and the available volume at which the spectrally resolved wave can be efficiently harvested. In this manuscript, on the other hand, we show that the “trapped rainbow” effect occurring in a defect free bulk structure can lead to considerable higher local field intensities by employing 3D PhC bulk modes with large modal volumes.

## Results

### Chirped 3D woodpile photonic crystal structure

**Figure 1:**
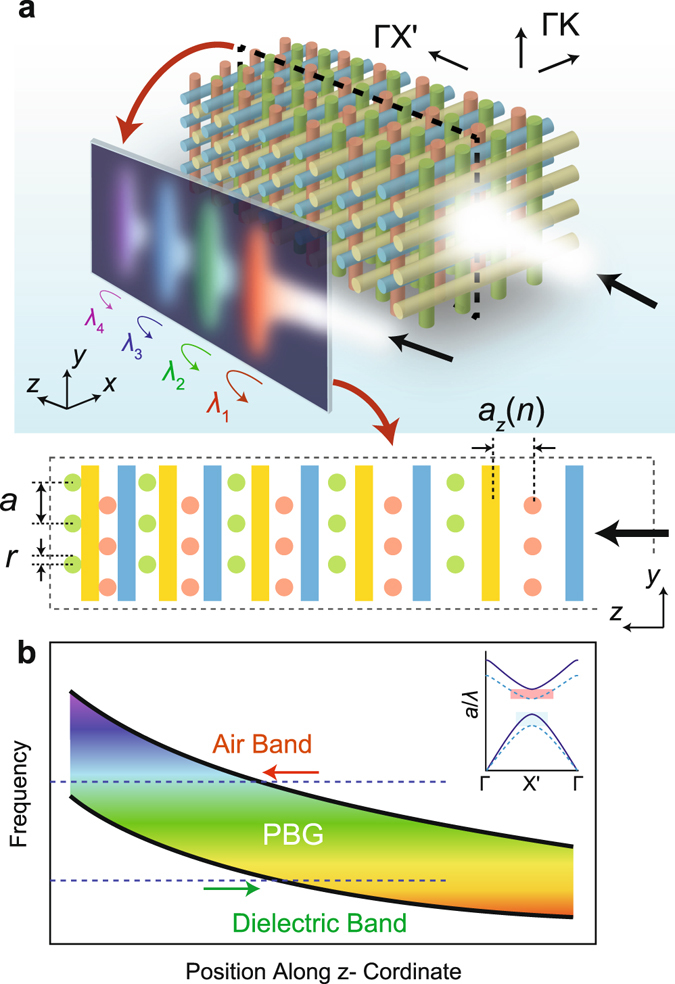
Proposed 3D PhC configuration for rainbow trapping. (**a**) Schematic illustration of the chirped woodpile PhC structure. For better visualization the number of periods of the illustrated PhC structure is reduced. The lower inset depicts the cross sectional view of the structure in the *yz-* plane. (**b**) The variation of the PBG along the propagation direction is representatively depicted. Due to the gradual variation of the layer-to-layer distances, the incident wave will be spatially separated into its frequency components along the propagation direction, thus forming a “trapped rainbow”, as it is representatively shown as a cross sectional view on the lower left of (**a**). The upper right inset in (**b**) representatively shows the change of the dispersion curves in the propagation direction, where the layer-to-layer distance of the solid black dispersion curve is smaller than that of the dashed blue dispersion curve. The black arrows reveal the propagation direction of the incoming wave.

We consider a 3D woodpile PhC composed of cylindrical alumina (Al_2_O_3_) rods with a radii and height equal to $r=\frac{\sqrt{2}}{8}a$ and *h* = 8.463*a*, respectively, where *a* is the in-plane period. The Al_2_O_3_ rods have a dielectric constant equal to *ε*_rod_ = 9.8 at microwave frequencies. The general schematics and the main operational principle of the proposed configuration is shown in Fig. 1(a). As mentioned above, the period in the stacking direction is continuously varied. We consider a slow (adiabatic) variation of the PhC period, which allows to consider local dispersion relation of it at every layer^24^. In result, the frequency of the PBG becomes smoothly varying along the propagation direction, as shown in Fig. 1(b). Therefore, a wave entering the structure will gradually slow down inside the PhC as its frequency will progressively approach the edge of the local PBG. Once the PBG is reached, the wave will “stop”, i.e. become temporarily localized, and afterwards will propagate back. Since the local PBG is distinct for every spatial position, each frequency component will be localized at different positions along the PhC, as illustrated in the left inset of Fig. 1(a).

### Dispersion analysis

**Figure 2:**
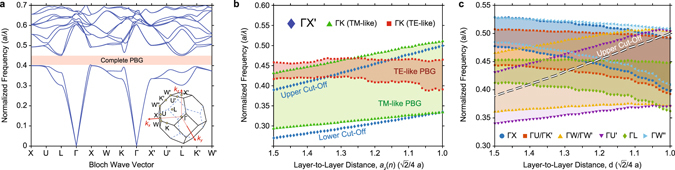
Bandstructure analysis of the woodpile PhC structure. (**a**) Numerically calculated band structure of the woodpile PhC with Al_2_O_3_ rods. The lower right inset depicts the first Brillouin zone of the woodpile structure. (**b**) The dependency of the layer-to-layer distance onto the longitudinal and transverse cut-off frequencies. The TM-like and TE-like PBGs in the transverse directions are highlighted with light green and red colored areas. (**c**) The PBG variations for other directions. The dashed white line indicates the upper cut-off frequencies in the Γ- X′ direction.

A detailed numerical analysis is performed to deduce the structure parameters. Preliminary, for a periodical woodpile PhC with a constant layer-to-layer distance $d=\frac{\sqrt{2}}{4}a$ (*i*.*e*. the rods from the consecutive layers just touch one another) the local band structure is numerically calculated with the plane-wave expansion method^38^ and the result is shown in Fig. 2(a). The calculated band structure reveals a complete PBG with a gap/mid-gap ratio (the ratio of gap width to the central frequency of the gap) of 11%, while the gap/mid-gap ratio of the partial PBG in the propagation direction Γ-X′ (stacking direction) is equal to 39%. Moreover, the frequencies at the bottom and top of this partial PBG are defined as the lower and upper cut-off frequencies and their variation with respect to the layer-to-layer distance is plotted in Fig. 2(b). Despite their similarity in terms of their layer-to-layer distance relations, the two cut-off regions differ in an important respect: To obtain a local PBG at the lower/upper bands, the layer-to-layer distance should be increased/decreased; respectively (see upper left inset of Fig. 1(b)). Consequently, the direction of the gradual layer-to-layer distance variation (whether it decreases or increases along the propagation direction) depends on the operational frequency region: The upper/lower cut-off regions require a decreasing/increasing layer-to-layer distance, respectively (indicated by red and green arrows in Fig. 1(b)). Apart from this apparent distinction, another difference between the two cut-off regions becomes evident if the partial PBGs in other directions are examined. The PBGs in the Γ-K direction, which corresponds to the direction along the rod axes are superimposed on Fig. 2(b). Unlike the Γ- X′ direction, the wave propagation in the Γ-K direction is nondegenerate for modes with polarization vectors along the rod axis and along the stacking direction, which we will refer to as the transverse electric-like (TE-like) and transverse magnetic-like (TM-like) modes, respectively. As can be seen from Fig. 2(b), the TM-like PBG comprises the whole upper cut-off region in the examined layer-to-layer distance range, whereas the TE-like PBG encloses only a portion of the examined range. On the other hand, for all utilized layer-to-layer distances, the lower cut-off region lies outside the coverage of both PBGs, which indicates that in the case of vertically scattered light, the corresponding structure based on the lower cut-off operation will be vulnerable to radiation losses. Furthermore, the PBG variations in the same range for propagation directions corresponding to the X, U, U′, K′, W, W’, W″, and L symmetry points^35^, whose positions in the Brillouin zone are shown on the lower right inset of Fig. 2(a), are calculated and given in Fig. 2(c). For directions with nondegenerate modes, the complete PBGs comprising simultaneously both TE-like and TM-like PBGs have been taken into account. Consequently, the local dispersion analyses in Fig. 2(b) and (c) imply that a complete confinement is not possible, since modifying the layer-to-layer distances will further break the low rotational symmetry of the lattice structure^39^, which in turn will increase the structural anisotropy. Nevertheless, a close inspection of the PBG variations reveals that for the layer-to-layer distance range $\{1.15,1.35\}\ast \frac{\sqrt{2}}{4}a$, the localized mode near the cut-off region is still confined in most directions.

Following this approach, it is reasonable to vary the layer-to-layer distances of the PhC structure from $1.35\ast \frac{\sqrt{2}}{4}a$ to $1.15\ast \frac{\sqrt{2}}{4}a$. However, one must take into account that the direct coupling into the slow light mode from the air region is problematic due to the mode mismatch at the air- PhC interface^40, 41^. Furthermore, in order to avoid photon tunneling into the air cladding, one needs additional layers at the end of the structure. These two requirements can be both satisfied by increasing the layer-to-layer distance interval further including $\{1.00,1.50\}\ast \frac{\sqrt{2}}{4}a$. In this way, the wave enters the structure with a wave vector away from the slow light region and the leakage due to the large penetration depth of the slow light modes is suppressed. On the other hand, since the operational frequency region is at the upper cut-off region, the layer-to-layer distance should decrease along the propagation direction, as discussed previously. Moreover, the number of rods in each layer is chosen to be 7, as it is restricted by the length of the Al_2_O_3_ rods (*h* = 8.463*a*). The number of layers, on the other hand, is chosen to be 30, to create a smoothly varying chirp function. In this case, the local layer-to-layer distance between the *n*th and (*n* + 1)th layer is equal to ${a}_{z}(n)=[(42.5-0.5n)\ast \frac{\sqrt{2}}{4}a]/28$. Here, we note that an important criterion that should be satisfied in adiabatic structures (to reduce the back-reflections) is the Wentzel-Kramers-Brillouin approximation^18^, which requires

1 $${\delta }_{n}=\frac{{k}^{-1}(n+1)-{k}^{-1}(n)}{{a}_{z}(n)}\ll 1$$

where *δ*_*n*_ is the adiabatic parameter between the nth and (*n* + 1)th layer and *k*(*n*) is the local wave vector at the *n*th layer. To reveal whether the adiabaticity condition is satisfied for the proposed PhC structure, the local wave vector distribution was obtained numerically for the frequency 7.91 GHz (which is predicted numerically to localize at the 24th layer), by calculating the dispersion curve for all intermediate periods. By inserting the obtained wave vector variation and the *a*_*z*_(*n*) variation into equation (1), the adiabatic parameter has been evaluated to be in the range of 0.005 and 0.009, which already satisfies equation (1). Possible solutions to further decrease the adiabatic parameter (and, thus, to decrease the back-reflections) could be increasing the number of layers or decreasing the chirping interval. However, the mode coupling mismatch at the slow light regime^40, 41^ should be further taken into account when tailoring the latter one, as was discussed above.

### Time-domain analysis

**Figure 3:**
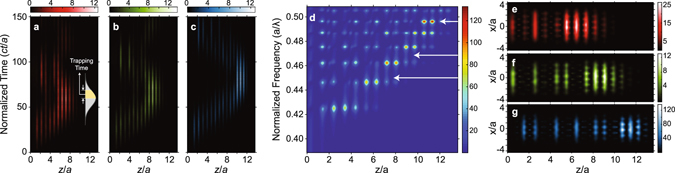
Instantaneous and steady-state field analysis. Time evolution of a Gaussian pulse propagating inside the 3D PhC (along the z- propagation direction, at the center of the *xy*- plane), for three frequencies: (**a**) *a*/*λ* = 0.450, (**b**) *a*/*λ* = 0.468 and (**c**) *a*/*λ* = 0.496. (**d**) Broadband steady-state field intensity distributions at the same propagation line. Steady-state field distributions obtained at the *xz*- plane of the center of the *y*- direction for the normalized operational frequencies equal to (**e**) *a*/*λ* = 0.450, (**f**) *a*/*λ* = 0.468 and (**g**) *a*/*λ* = 0.496. The magnitude of the field intensities are normalized by dividing the calculated intensities to the intensity produced by the source at the specific frequencies. The distances in the z- propagation direction are given with respect to the position of the source.

To verify the propagation characteristics, the proposed structure was modeled in a 3D grid by employing commercially available software^42^ based on the finite-difference time-domain (FDTD) method. Perfectly matched layer (PML) boundary conditions were used to terminate the computational domain and a TM polarized (electric field is in the *y*- direction) Gaussian pulse with a pulse length equal to *ct*/*a* = 30 in normalized time units was launched. The source was placed at a distance of 0.2*a* in front of the structure. Fig. 3(a–c) show the electric field intensities, calculated along the propagation (*z*-) direction, at the center point of the *xy*- plane at each time frame for normalized central frequencies *a*/*λ* = 0.450, 0.468 and 0.496, respectively. Figures 3(a–c) suggest that the PhC exhibits distinct localization regions for each frequency. More precisely, the incoming wave is slowed down until it reaches its localization region (or turning point) and is then trapped for a finite time interval before it starts propagating backwards. If the trapping time is defined as the time duration of the electric field intensity decaying from its maximum value until its 1/*e* ^19^, then the normalized trapping times for Figs 3(a–c) would be obtained as *ct*/*a* = 18.9, 20.3 and 24.1; respectively. Further inferences can be made from the time evolution of the pulses, if one pays attention to the change of the magnitude of the field intensity. At the localization region the intensity increases, since the local energy density is expected to be inversely proportional to the group velocity^20, 21, 43^. Furthermore, to obtain a broadband analysis of the field enhancement, the steady-state electric field intensity was numerically calculated at the same region (along the z- propagation direction, at the center of the *xy*- plane), and is shown in Fig. 3(d). If the turning point is defined as the spatial position where the intensity becomes maximum, then a nearly linear increase of the position of the turning point is observed, which originates from both facts that the upper cut-off region is nearly linearly dependent on the layer-to-layer distance (see Fig. 1(b)) and that the chirp function is varying linearly. One interesting observation from Fig. 3(d) is that the intensity enhancement is highly sensitive to the operation frequency. In particular, for the normalized operation frequency range *a*/*λ* = {0.42, 0.50} the maximum normalized intensities occurring at the localization regions deviate between 8 and 132. We attribute this deviation to the oscillations occurring between the turning point and the entrance of the structure. For instance, Fig. 3(a–c) suggest that the intensity enhancement is nearly equal for these frequencies, which is expected since the nearly linear cut-off variation will induce similar slowdowns for different frequencies, even though their steady-state intensity enhancements are much diversified, as indicated with white arrows in Fig. 3(d). The main difference between these two cases is that the steady state analysis is the response of a continuous source excitement, which comprises multiple enhancement factors such as slowing down and multiple interferences of the reflected waves from the turning point and at the entrance of the structure; whereas the time evolution analysis is the response of a source with a finite temporal pulse width, which includes only the slowing mechanism in terms of the intensity enhancement. We therefore believe that the oscillatory behavior of the intensity enhancement stems from the peculiar relation between the optical path and the local wave vector distribution. In this regard, for a specific frequency, the local wave vector distribution leads to such a local wavelength distribution that it will encounter constructive interferences within the optical path (which is also unique to the operational frequency). In other words, the slowing down of the wave will be accompanied by a structural resonance, which in result give rise to an additional enhancement factor. Such frequencies correspond to the narrowband peaks in Fig. 3(d) and one can infer from this figure that the spectral distance between these peaks decreases as the operational frequency increases. This is also reasonable, since increasing the frequency will cause the wave to localize and reflect further away from the entrance of the structure and to enter the structure with a wave vector away from the cut-off region, compared to lower operation frequencies. This will have the consequence that the wavelength distribution will initiate with lower spatial wavelengths as the wave enters the structure^21^, in which case it will be enough to increase the operational frequency less compared to lower frequencies, to match up the next constructively interfered frequency.

Furthermore, the steady state electric field intensities along the *xz*- plane at the middle of the structure in the *y*- transverse direction were calculated and are shown in Fig. 3(e–g) for the normalized operation frequencies equal to *a*/*λ* = 0.450, 0.468 and 0.496; respectively. It follows from these figures that the intensity enhancements differentiate that from the pulse excitations, as expected. Furthermore, additional localization regions occur apart from the localization region at the turning point, since the abovementioned resonator effect will give rise to a standing wave within the respective optical path. Nevertheless, the local energy density at the turning points will be higher than at the additional localization regions, as the slowing down will become maximum at these points^21^.

**Figure 4:**
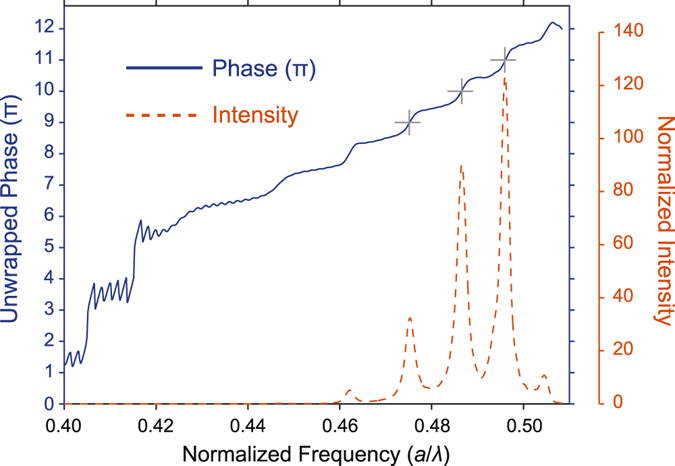
Accumulated phase and intensity enhancement relation. Superimposed phase and normalized intensity spectrum. The unwrapped phases are calculated with respect to the phase at the entrance of the structure. The gray cross markers indicate the locations of the intensity peaks.

To quantitatively evidence our interpretation regarding the additional enhancement mechanism described above, we calculated the phase of the different frequency components at a particular position. Figure 4 shows the unwrapped phase of the electric field in the *y*- direction at the longitudinal position equal to 10.55*a* with respect to the front face of the structure. Moreover, the normalized intensity spectrum at the same spatial position is shown in Fig. 4. It follows that for the frequencies, at which the intensities are locally maximum, the phases are integer multiples of π. This is in accord with our abovementioned interpretation of the large enhancement factors, since a constructive interference between a specific path requires also the same amount of phase shift^44^.

**Figure 5:**
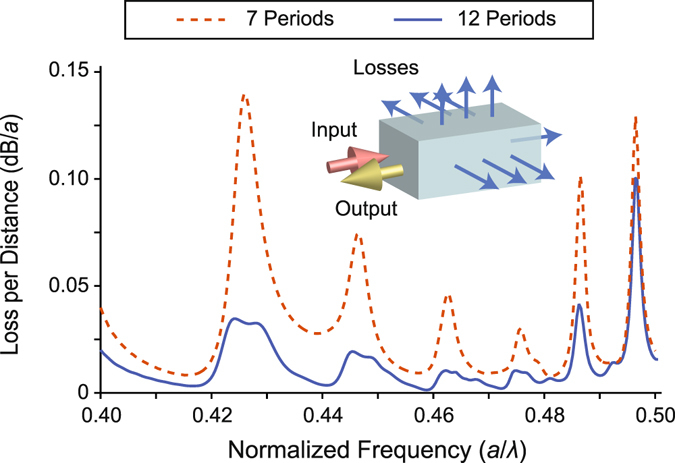
Lateral loss analysis. Superimposed loss spectra of the PhC structure, with 7 and 12 transverse periods is shown. Note the oscillatory behavior, which is a direct consequence of the resonant propagation of the wave between the localization region and the input side of the structure.

Further numerical analyses were carried out to reveal the quantitative amount of propagation losses, which occur due to the finite transverse dimensions of the structure. The propagation losses were characterized by calculating the reflected power, by taking into consideration that in the case of a lossless propagation all incident power should be eventually reflected back after it is localized. The calculated reflected power is then normalized to the incident source power to give quantitative information about the loss spectra. Strictly speaking, since every frequency component will travel a different distance, it is more physically meaningful to normalize the loss to the distance travelled by the wave for every frequency component. The travelling distance has estimated as the twofold of the distance between the entrance of the structure and the turning point, by taking into account the forward and backward propagation distances. Therefore, for a more accurate analysis one must take this fact into account. However, in the present calculations we ignore this fact and define the travelling distance as an effective distance travelled by the wave, excluding the additional round trips. Following this direction, the loss spectra is calculated for 7 (original structure) and 12 (extended structure) transverse periods and are superimposed in Fig. 5. It can be observed from this figure that for the normalized frequency interval *a*/*λ* = {0.44, 0.48} the propagation losses are lower than the rest of the operational frequency spectrum, which is expected as the TE-like transverse PBG does not compromise outside this interval, as was discussed. Another conclusion that can be drawn from Fig. 5 is that increasing the number of the transverse periods reduces the losses, as expected. It is worth noticing that the narrowband enhancement peaks in Fig. 3(d) matches well with the loss peaks in Fig. 5. As above discussed, the narrowband enhancement peaks arises from multiple roundtrips inside the structure, which lies also in the origin of the enhanced losses.

### Experimental test

**Figure 6:**
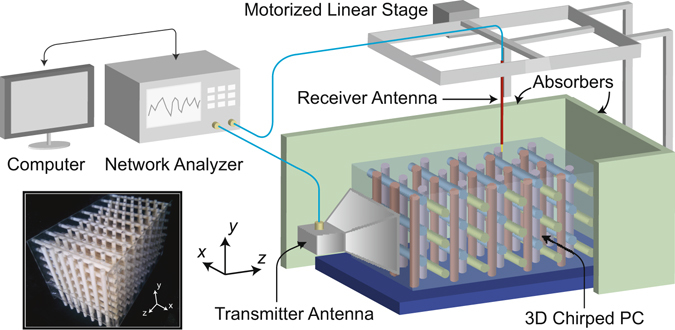
Microwave experimental setup. Schematic illustration of the experimental setup is shown. The lower left inset depicts the photographic view of the constructed woodpile PC.

**Figure 7:**
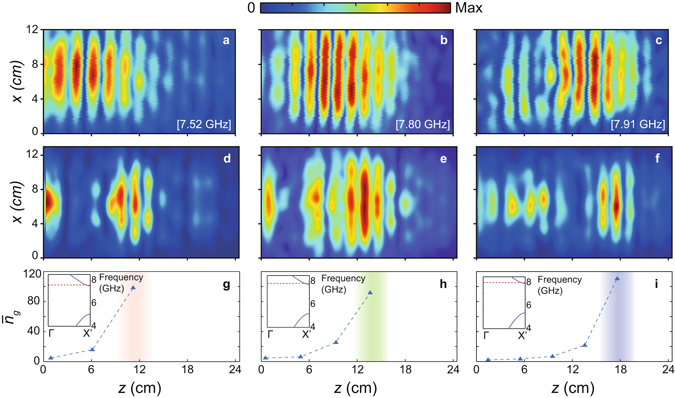
Steady-state field and group index measurements. (**a–c**) Experimentally measured and (**c–e**) numerically calculated steady state electric field distributions at the top xz- surface of the structure for frequencies (**a–d**) 7.52 GHz, (**b–e**) 7.80 GHz and (**c–f**) 7.91 GHz are shown. Experimentally measured average group indices for frequencies (**g**) 7.52 GHz, (**h**) 7.80 GHz and (**i**) 7.91 GHz are given. The colored areas denote the localization regions. The upper left insets show the calculated bandstructure for layer indexes equal to 16, 20 and 24; which are determined as the localized positions of the frequencies 7.52 GHz, 7.80 GHz and 7.91 GHz, respectively. The dashed red lines reveal the operational frequencies.

To verify the propagation characteristics experimentally in the microwave regime, the proposed structure was constructed from absorption free Al_2_O_3_ rods (see Fig. 6). Prior to performing the experimental analyses, the coupling efficiency has been numerically estimated to be in the range of 65–71% and experimentally in the range of 62–74%. The numerical estimation was based on the termination of the simulations at the specific time step where the initial reflections due to air-PhC coupling mismatch have been completely gone through the reflection monitor. Experimentally, on the other hand, a strong microwave absorber was placed inside the PhC structure to similarly avoid the chirping reflections and to account only for the reflections arising from the coupling mismatch. The discrepancy between the experimental and numerical estimations stems mainly from the additional reflections and interferences inside the receiver antenna’s metallic aperture and to the non-ideal absorption (approximately 95%) of the microwave absorber. In contrast to the calculations, in our experimental setup (see Fig. 6) we could scan the electric field distribution only at the top surface of the structure. The monopole antenna was kept parallel to the *y*- axis and the tip of the antenna was placed 2 mm above the top of structure. A motorized linear stage was used to move the monopole antenna with 1 mm lateral resolutions in the *x*- and *z*- directions, as shown in Fig. 6. In this way, the *y*- component of the electric field at the top surface of the structure was measured and shown in Fig. 7(a–c) for frequencies equal to 7.52 GHz (*a*/*λ* = 0.450), 7.80 GHz (*a*/*λ* = 0.467) and 7.91 GHz (*a*/*λ* = 0.473), respectively. On the other hand, the electric field distribution was numerically calculated at the same surface and is in Fig. 7(d–f) for frequencies equal to 7.52 GHz, 7.80 GHz and 7.91 GHz, respectively. Comparing Fig. 7(a) with Fig. 7(d), Fig. 7(b) with Fig. 7(e) and Fig. 7(c) with Fig. 7(f), we note that the turning points in the numerical and experimental cases are in good agreement. We attribute the discrepancy in the field distributions between the two cases to the difference of the experimental and numerical source beam shapes.

To experimentally evidence that the group velocity of the propagating wave converges to zero towards the localization regions, we performed a series of time delay measurements^45^. Figure 7(g–i) show the measured average group indices ${\bar{n}}_{g}$ for the frequencies equal to 7.52 GHz, 7.80 GHz and 7.91 GHz, respectively. We find that in all three cases ${\bar{n}}_{g}$ gradually increases towards the localization region. At these regions, ${\bar{n}}_{g}$ varies between 90 and 110, which verifies the slow light originating localizations^20^. We should note that in theory one expects to obtain zero group velocity at the localization regions, however there are two major factors that prevent obtaining such a measurement. The first one is the lateral losses occurring at the localization region, which impose an additional decay rate to the system and cause deviation of the group velocity from zero. The second one, which is rather a practical issue, is the difficulty to obtain the time delay measurements continuously along the longitudinal direction. As was said above, due to the 3D periodicity of the structure, the time delay measurements had to be taken at discrete positions which transforms the *τ*_*d*_ = ∂*φ*/∂*ω* differential relation into a finite difference approximation. To further reveal that the localized modes approach the band edge regions, the bandstructure in the propagation Γ-X′ direction for the instantaneous a_z_ periods at the localization regions were numerically calculated (see upper left insets of Fig. 7(g–i)). One can infer from these figures that the localized modes are indeed close to the band edge region and, thus, are expected to be slowed down and be localized.

**Figure 8:**
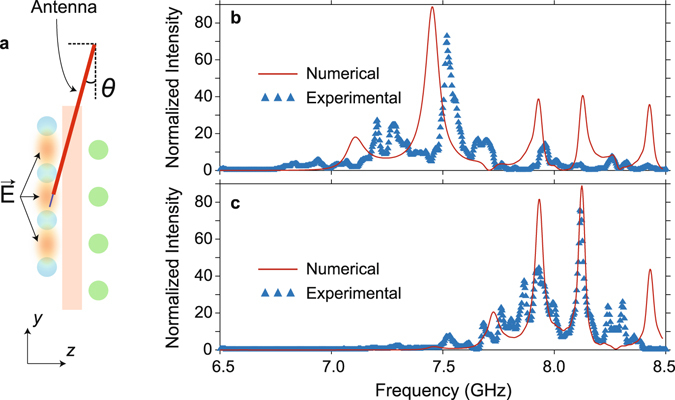
Field enhancement measurements. (**a**) Schematic description of the measurement of the electric field intensity inside the structure. The numerically calculated and experimentally measured electric field intensity spectra, obtained at the center of the *xy*- plane and at a distance equal to (**b**) 9.25 cm and (**c**) 17.15 cm in the *z*- direction with respect to the input of the structure are superimposed.

In view of the fact that the field distribution obtained at the top surface of the structure would not give any implication about the intensity enhancement, we further measured the electric field intensity spectrum at single points inside the structure (see Fig. 8(a)) to estimate the enhancement factors. Accordingly the normalized intensities were obtained at the center of the structural heights in the *x*- and *y*- directions and at a distance in the propagation *z*- direction equal to 5.15*a* = 9.25 cm and 9.55*a* = 17.15 cm with respect to the input side of the structure, and are superimposed with the regarding numerical calculations in Fig. 8(b,c), respectively. One can infer from these figures that the experimentally measured maximum intensities are around 72–74 for both cases, whereas the numerically calculated intensities are around 88–89. Moreover, one can observe that there are some spectral peak shifts and intensity differences between the numerical and experimental cases. We attributed these discrepancies to the structural fabrication errors, the non-uniform detection efficiency of the monopole antenna, the modified component of the detected electric field due to antenna tilting and the positional error of the detected area. Nevertheless, the experimental results verified that the wave can be enhanced and localized at various positions depending on its frequency.

## Discussion

In summary, a chirped woodpile PhC, whose layer-to-layer distances are gradually decreased along the propagation direction, has been numerically and experimentally demonstrated to slow down and finally to trap and enhance different frequency components at different spatial positions. It has been argued that the Fabry-Perot interferences occurring between the turning points and the entrance of the structure together with the adiabatic slowing mechanism can lead to intensity enhancements close to two orders of magnitude. An experimental realization at the microwave regime verified the operation principle, and we further note that the proposed structure is also feasible for current nanofabrication technologies, such as electron-beam lithography^46^ or direct laser writing techniques^47, 48^, due to its simple layer-by-layer architecture and its robustness against fabrication disorders^34, 35^. Furthermore we showed that the propagation losses can be suppressed, owing to the 3D periodicity of the structure. Such a phenomenon could be exploited to realize nonlinear optical devices, broadband photon harvesting systems, wavelength division multiplexing devices and optical buffers. We should note however, that the light enhancement phenomenon in the proposed configuration should not be considered as a permanent storage of a ‘trapped rainbow’^7^, but rather a temporary localization, due to the finite trapping time caused by the reflection at the turning points^25, 49^. It should be also noted that although high group indices up to 110 have been measured, a zero group velocity near the localization region could not be obtained due to additional out-of-plane losses and experimental limitations.

## Methods

### The experimental setup and the construction of the 3D woodpile PhC structure

Plates made of Plexiglas were employed to embody the constructed 3D PhC structure. By employing a laser engraving machine, holes were drilled inside the Plexiglas plates at the exact positions where the Al_2_O_3_ rods should be placed. The refractive index of Plexiglas has been measured via Bragg condition^50^ to be equal to 1.59 and numerical analyses revealed that placing the Plexiglas plates does not change the results significantly. The structural parameters are same as in the numerical calculations, where the in-plane period is set equal to *a* = 17.96 mm. The experimental setup (see Fig. 6), consists of a standard horn antenna, a monopole coaxial antenna and a vector network analyzer (Agilent 5071 C ENA). The horn antenna has an aperture size of 12.5 cm and 9.5 cm in the *x*- and *y*- directions, respectively, and is used to inject electromagnetic waves into the structure with electric field polarized in the *y*- direction, whereas the monopole antenna is used to detect the radiated electromagnetic waves. Furthermore, the experimental setup was covered with microwave absorbers to minimize the noise due to environmental reflections.

### Measurement of the time delay

The time delay is a measure of the photon transit time and is defined as *τ*_*d*_ = ∂*φ*/∂*ω*, where *φ* is the phase difference between the input and output port of the vector network analyzer and *ω* is the operational angular frequency. Since the 3D periodicity of the woodpile PhC structure prevents to take a continuous spatial measurement along the longitudinal *z*- direction, we injected the receiver antenna inside the PhC structure with a tilting angle of 16° with respect to the *y*- axis until the center of the structural height at specific points. The difference of the time delays measured at these points have been divided to the corresponding point-to-point spatial distance to obtain the average group velocity, which is then divided to the speed of light in free space to estimate the average group index ${\bar{n}}_{g}$.

### Measurement of the intensity enhancement

The monopole antenna was inserted into the structure until the tip of the antenna reaches the center of the structural height in the *y*- direction. Keeping in mind that the monopole antenna should be parallel to the polarization axis of the injected electric field, the fact that the electric field is localized mostly between the rods that are perpendicular to the polarization axis inhibits the monopole antenna to be inserted parallel to the polarization axis. Therefore, as shown in Fig. 8(a) the monopole antenna was inserted from the neighboring layer inside and was tilted towards the region of interest with an angle of 16°. The measured electric field intensity was then normalized by the intensity measured with the same tilt angle at the center of the aperture of the transmitter horn antenna.
